# Interaction of Ras Binding Domain (RBD) by chemotherapeutic zinc oxide nanoparticles: Progress towards RAS pathway protein interference

**DOI:** 10.1371/journal.pone.0243802

**Published:** 2020-12-16

**Authors:** Elza Neelima Mathew, Miranda N. Hurst, Baolin Wang, Vaibhav Murthy, Yuntao Zhang, Robert K. DeLong

**Affiliations:** 1 Department of Anatomy and Physiology, College of Veterinary Medicine, Nanotechnology Innovation Center of Kansas State, Kansas State University, Manhattan, Kansas, United States of America; 2 Molecular Biophysics, Kreiger School of Arts and Sciences, Johns Hopkins University, Baltimore, Maryland, United States of America; 3 Center for Retrovirus Research, College of Veterinary Medicine, Ohio State University, Columbus, Ohio, United States of America; University of Southern Denmark, DENMARK

## Abstract

Zinc oxide (ZnO) NP is considered as a nanoscale chemotherapeutic. Thus, the drug delivery of this inorganic NP is of considerable importance. Ras mutations are common in cancer and the activation of this signaling pathway is a hallmark in carcinoma, melanoma and many other aggressive malignancies. Thus, here we examined the binding and delivery of Ras binding domain (RBD), a model cancer-relevant protein and effector of Ras by ZnO NP. Shifts in zeta potential in water, PBS, DMEM and DMEM supplemented with FBS supported NP interaction to RBD. Fluorescence quenching of the NP was concentration-dependent for RBD, Stern–Volmer analysis of this data was used to estimate binding strength which was significant for ZnO-RBD (K_d_ < 10^−5^). ZnO NP interaction to RBD was further confirmed by pull-down assay demonstrated by SDS-PAGE analysis. The ability of ZnO NP to inhibit 3-D tumor spheroid was demonstrated in HeLa cell spheroids—the ZnO NP breaking apart these structures revealing a significant (>50%) zone of killing as shown by light and fluorescence microscopy after intra-vital staining. ZnO 100 nm was superior to ZnO 14 nm in terms of anticancer activity. When bound to ZnO NP, the anticancer activity of RBD was enhanced. These data indicate the potential diagnostic application or therapeutic activity of RBD-NP complexes *in vivo* which demands further investigation.

## Introduction

Ras is a protein along the cellular pathway between mitogen activated protein kinases and extracellular signal activated kinases (MAPK-ERK pathway) which has thus far eluded traditional drug discovery. There are three different human Ras genes–KRas, NRas and HRas, the mutation predominance of which varies with the cancer type [1]. Given the importance of Ras signaling pathways in many cancers, an elegant approach to target this would be the intracellular delivery of Ras binding domain (RBD). Enrichment of metal oxide nanoparticles via surface functionalization with tissuespecific biological or chemical agents makes it easier to navigate to the exact locus [2]. While RBD lacks the kinase domain of B-Raf, abolishing its ability to upregulate this pathway, this 149 amino acid portion of BRAF, is responsible for binding to RAS and thought to act as a decoy. However, RBD does not contain any hydrophobic sequence or membrane penetration sequence, thus requiring a vehicle for increased delivery to cancer cells. Therefore, it seemed plausible to interact RBD with zinc oxide nanoparticle (ZnO NP) and this novel approach might improve the drug delivery and anticancer activity [3, 4].

Physiologically-based zinc oxide (ZnO) is a highly sought after nanoparticle owing to its unique intrinsic and indigenous anticancer activity and has been shown to be selectively toxic to a variety of cancer cell types including cancerous T cells, human hepatocellular carcinoma (HepG2), human bronchial epithelial cells (BEAS-2B) and human lung adenocarcinoma (A549) cells [5, 6]. ZnO NP generates reactive oxygen species (ROS) which can also trigger cancer cells to undergo apoptosis [7, 8]. Intraperitoneal administration of ZnO has been associated with an increase in superoxide dismutase activity and total antioxidant status in male rats [9]. Its unique surface chemistry allows formation of ZnOH+ species which likely underlies its interaction to anionic membrane of cancer cells and its drug delivery activity [10, 11]. Recent work from our research group suggests ZnO NP inhibition of experimental melanoma [12] can be improved by its interaction to poly I:C RNA [13].

In this study, we comparatively characterized ZnO NP– 14 nm and 100 nm–association to RBD and their potential for RBD delivery and the anticancer activity associated therewith.

## Materials & methods

### Materials and equipment

ZnO NPs (size < 100nm) were purchased from Sigma–Aldrich (St. Louis, MO). Raf-1 RBD, GST (recombinant protein expressed in *E*. *coli*: purity > 50%) was purchased from EMD Millipore Corporation (California, USA). Dynamic light scattering and zeta potential measurements were performed using Malvern Zetasizer Nano ZSP (Worcestershire, UK). Fluorescence spectroscopy was conducted using SpectraMax^®^ i3x multimode microplate reader (Molecular Devices, California, USA). For SDS-PAGE, RunBlue™ precast gels were purchased from Expedeon Ltd. (Cambridge, UK) and GelCode™ Blue safe protein stain was purchased from ThermoFisher Scientific (Waltham, MA, USA). Gel imaging was conducted using Gel Doc™ XR+ gel documentation system (BIO-RAD, California, USA).

B16-F10 mouse melanoma cells and Dulbecco’s Modified Eagle’s Medium (DMEM) with high glucose and L-glutamine for cell culture were purchased from ATCC^®^ (Virginia, USA). Phosphate buffered saline (PBS) was purchased from Corning^®^ (Pennsylvania, USA). FITC Annexin V Apoptosis Detection Kit I was purchased from BD Biosciences (Maryland, USA).

### NP stock preparation

Nanoparticles (~1 mg) were washed twice in 70% isopropanol followed by 100% isopropanol. Washed nanoparticles were airdried overnight. The dry pellets were dispersed in 1 mL of HPLC grade water to make 1mg/mL stock solutions.

### *Zeta* potential measurement

Samples were prepared by adding 20μL of nanoparticle stock solution (1mg/mL) in the 980μL of different dispersants namely 1) HPLC grade water, 2) DMEM with fetal bovine serum (FBS), 3) DMEM without FBS and 4) PBS. Afterwards, the samples were analyzed at a volume of 1 mL in a capillary cell cuvette (Malvern) on a Malvern Zetasizer Nano Range Dynamic Light Scattering instrument.

### Fluorescence spectroscopy

Samples were prepared at a final volume of 200 μL, and transferred to a black 96 well microplate (Midsci). Fluorescence spectra were taken at an excitation wavelength of 350 nm using the SpectraMax^®^ i3x multimode microplate reader with steps of 2 nm and a read height of 1 mm. The binding constant was evaluated by analyzing the fluorescence quenching with the Stern–Volmer equation [13, 14].

### SDS–PAGE

RBD-NP samples were prepared by combining 2μg of RBD (1 μL of 2μg/μL stock solution) and 5μg of ZnO NP (5μL of 1mg/mL stock solution). The RBD-NP samples were incubated at room temperature for 15 minutes with gentle shaking. Following incubation, the samples were subjected to sodium dodecyl sulfate polyacrylamide gel electrophoresis (SDS-PAGE) at 100V for 2 h hours and 30 minutes. BEPAR Bullseye pre-stained protein ladder (10–180 kDa) was used as the reference. [*Loading sample composition*: Sample loading buffer - 5μL; Sample (NP and BSA—5μL each) - 10 μL; HPLC grade water—5 μL; Total—20 μL.

#### Running buffer composition

RunBlue™ 20X buffer—50 mL; Deionized water—950 mL; [Total - 1L]

The protein bands were visualized using GelCode™ Blue Safe Protein Stain. The gel was imaged using Gel Doc™ XR+ gel documentation system and analyzed with Image Lab™ software.

### Determination of hydrodynamic size of ZnO NP in cell culture medium over time

The size of ZnO NPs in cell culture medium was determined using dynamic light scattering analysis. In brief, 20μg/ml solutions of ZnO NP (14 nm and 100 nm) were incubated at 37°C with shaking for 72 h. Size measurements were taken at 0h, 1h, 2h, 4h, 8h, 12h, 24h, 48h and 72h in duplicates on a Malvern Zetasizer Nano Range Dynamic Light Scattering instrument.

### Determination of dissolution of ZnO NPs in cell culture medium

The concentration of ZnO NPs in the cell culture medium was determined using ICP-MS analysis [15]. In brief, 20μg/ml solutions of ZnO NP (14 nm and 100 nm) were incubated at 37°C with shaking for 72 h. Size measurements were taken at 0h, 1h, 2h, 4h, 8h, 12h, 24h, 48h and 72h in duplicates on a Malvern Zetasizer Nano Range Dynamic Light Scattering instrument. Following size measurement, the samples were centrifuged at 14,000 rpm for 1 minute. 100 μl of the supernatant containing ZnO NP was digested using 1 ml of 70% nitric acid (HNO3). The digestion was performed in a hot water bath at 60°C for 45 minutes. Following digestion, all the digests were diluted by addition of 9 ml deionized water. Zinc ion concentration was measured on a PerkinElmer NexION^®^ 350D ICP-MS using Syngistix™ software (Shelton, CT, USA).

### Spheroid assay

HeLa cells were plated at 5.0x10^3^ cells per well in *InSphero* GravityTRAP ULA plates (*PerkinElmer*, *Cat# ISP-09-001*) following the manufacturer’s protocol. Spheroids were allowed to form for 2 days, after which the NP treatment was applied (10 to 20 ug/mL) and the cells were imaged by light microscopy and by fluorescence microscopy after staining with Invitrogen live/dead green/red fluorescent staining kit.

### MTT assay

B16F10 cells were plated on a 96-well plate at 5,000 cells per well and were allowed to attach overnight. The next day, they were treated with 1, 5, 10, 20, 50, or 100 μg/mL of the ZnO NP (14 and 100 nm). After 24 hours with the above treatments, cells were rinsed with PBS and MTT reagent was added. After incubation with the MTT reagent for 5h, DMSO was used to dissolve the formazan crystals and the absorbance was read at 562 nm using a Synergy H1 Hybrid Multi-Mode Reader (BioTek) to estimate cell viability.

For testing the effect of RBD bound NPs, 20 μg/mL of ZnO NP-100 nm was used with a final concentration of 25μg/mL RBD bound. An additional group of RBD alone was tested, also at a final concentration of 25 μg/mL.

Untreated cell culture medium served as the blank. Untreated cells were used the control. The experiment was performed in quadruplicate wells.

### Apoptosis assay

B16F10 cells were plated on 8 –well chamber plates at 3 × 10^4^ cells per well and allowed to establish overnight. Cells were treated with washed nanoparticles at a concentration of 0.05 mg/mL. Cells were stained using FITC Annexin V Apoptosis Detection Kit I and imaged at 0 and 12 hours using an Olympus CKX41 Inverted Microscope with Olympus SC100 camera and Analysis getIT™ imaging Software. As the positive control, 50μM H_2_O_2_ was used.

## Results and discussion

### Confirming interaction of RBD to ZnO NPs

Many proteins contain charged amino acids which can facilitate interaction to the surface of nanoparticles. Such ionic interactions and close association of the protein to the surface of the nanoparticle causes a change in its surface charge and this can be measured by zeta potential analysis. As shown in Table 1, zeta potential measurements revealed a shift in the surface charge of ZnO NPs following incubation with RBD, suggesting interaction to the protein. The zeta potential here is reported as the average of the multiple peaks as the sample was polydispersed [16].

**Table 1:** Zeta potential measurements of ZnO NP-100 nm in response to RBD binding.

	Solvent	NP alone	NP + RBD
Zinc oxide (ZnO-100nm)	HPLC grade water	-17.86±0.3628	-17.86±0.5307
	PBS	-23.22±0.6807	-11.14±0.3709
	DMEM	-13.3±0.7797	-10.86±0.34
	DMEM+FBS	-6.91±0.1881	-8.152±0.4776

**Fig 1:**
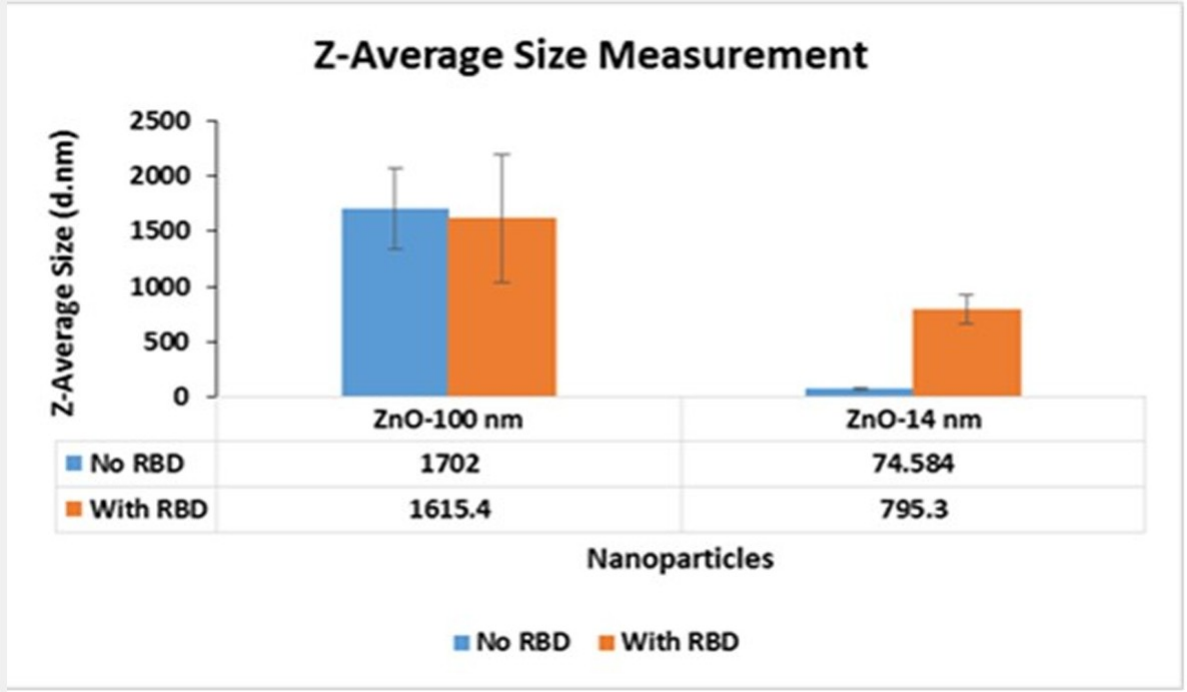
Size measurements of ZnO NP-100 nm and 14 nm.

Size variations both in the presence and absence of RBD was also compared with two differently sized ZnO NPs– 14 nm and 100 nm as shown in Fig 1.

The charge of RBD was estimated to be 7.3 at pH 7.0 using the online protein sequence analysis tool Protein Calculator v3.4 (Scripps Research, California, USA). Considering this fact, a positive shift was expected in the zeta potential of nanoparticles bound with RBD. With ZnO NP, such a positive shift was observed when dispersed in DMEM and PBS.

**Fig 2:**
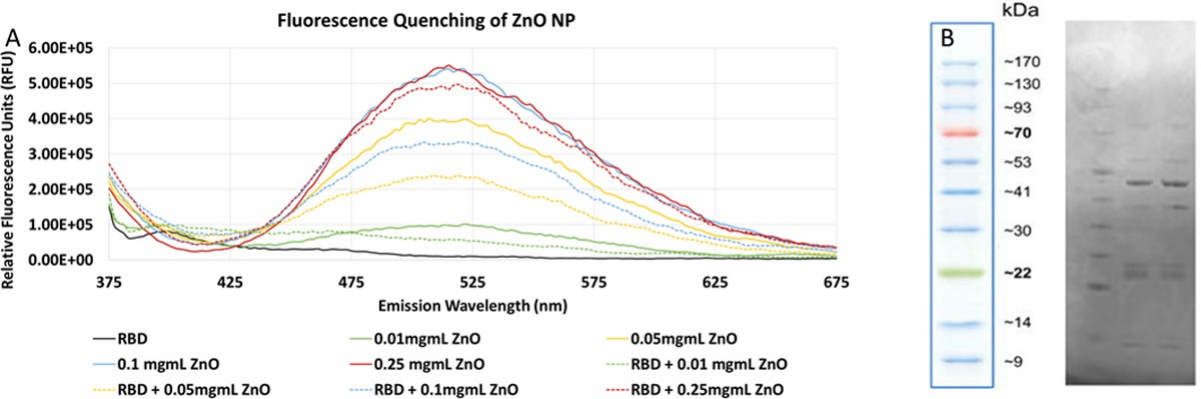
A. Fluorescence quenching of ZnO NP in the presence and absence of RBD measured at 350 nm further confirming interaction. B. SDS-PAGE analysis of RBD and ZnO nanoparticle combination: Lane 1 –Protein Ladder Lane 2 –RBD alone Lane 3 –RBD and ZnO NP combination.

Determination of fluorescence quenching by spectroscopy is a traditional and convenient method to assess protein interaction with nanoparticle [17]. The fluorescence spectra (Fig 2A) suggests strong association of RBD to ZnO. It also shows a dose dependency, with higher doses of ZnO NP showing higher fluorescence values, which is suppressed upon binding to RBD.

The suppression of the fluorescence with RBD present at equimolar concentrations of NP suggests interaction. Stern-Volmer equation was applied to calculate the dissociation constant and the number of binding sites as we have previous described for ZnO NP (12). These data are shown in Table 2 below. Accordingly, the association of RBD with ZnO NPs appeared to be strong.

**Table 2:** Dissociation constant of RBD complexed to zinc oxide (ZnO NP-100nm).

Nanoparticle	K_sv_ (1/mM)	n	K_d_ (mol/L)
Zinc oxide (ZnO NP-100nm)	13.087	1.3696, ~1	1.2306×10^−5^

K_sv_- Stern-Volmer dynamic quenching constant; n- number of binding sites; K_d_- dissociation constant

### Protein gel electrophoresis analysis

Protein gel electrophoresis is a standard method for studying protein interaction to NP. For example, an earlier study demonstrated the presence of transferrin and/or human serum albumin on the surface of polystyrene nanoparticles were effectively analyzed using SDS-PAGE [18]. SDS-PAGE was also used to compare the amount of proteins adsorbed on gold nanoparticles [19]. To further examine the interaction between RBD and the ZnO NP, the complexes were subjected to protein gel electrophoresis analysis following incubation at room temperature for 15 minutes. Elution of the protein from the particle demonstrated a banding pattern almost identical to that of the free protein control as seen in Fig 2B. suggesting that ZnO NP does not have any degradative effect on the protein.

### Physical stability of ZnO NP in biological media

**Fig 3:**
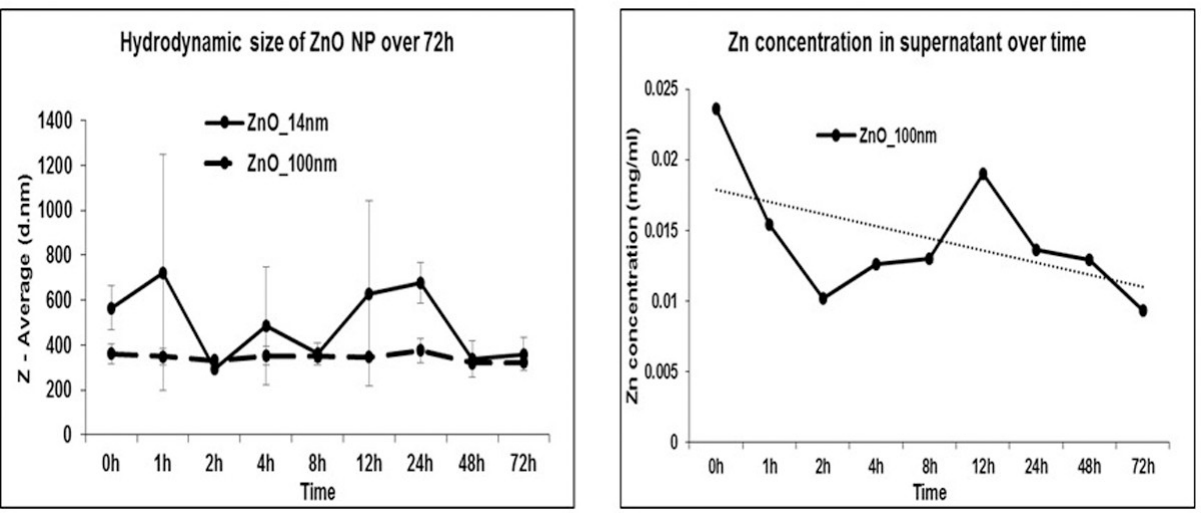
A. Hydrodynamic size of ZnO NPs (14nm and 100 nm) monitored over 72h time course. B. Zinc concentration in supernatant monitored over 72h time course.

It is important that NPs maintain their size stability when incubated in biological fluids. ZnO NPs (14 nm and 100 nm) were incubated in cell culture medium at 37°C and agitated on a shaker for 72 h and the effect on size measurements determined at 0h, 1h, 2h, 4h, 8h, 12h, 24h, 48h and 72h. As Fig 3A shows, ZnO NP (14 nm) showed variation in hydrodynamic size all through the time course following an irregular pattern. However, ZnO NP (100 nm) was found to maintain its hydrodynamic size and was quite stable and consistent with very little variation for the duration of the experiment.

### ZnO NP does not dissociate in cell culture medium

Current dogma suggests ZnO NP may disassociate into ions limiting its utility as in vivo drug carriers This property is thought to underlie its anticancer activity within the tumor microenvironment and cancer cells [20, 21]. However, there is a difference of opinion on the perception of particle dissolution as the major cytotoxicity mechanism of ZnO NP [22]. To address this, zinc concentration was monitored in the supernatants of ZnO NPs suspended in serum containing cell culture medium incubated at 37°C using ICP-MS. As evident from Fig 3B, zinc concentration in the cell culture medium supernatant followed a decreasing trend over time. This observation suggests that ZnO NP does not dissociate in normal physiological conditions. The cellular effects we see shall be attributed to ZnO NP itself, rather than zinc ions.

### Zinc oxide nanoparticles inhibit 3-D spheroids

**Fig 4:**
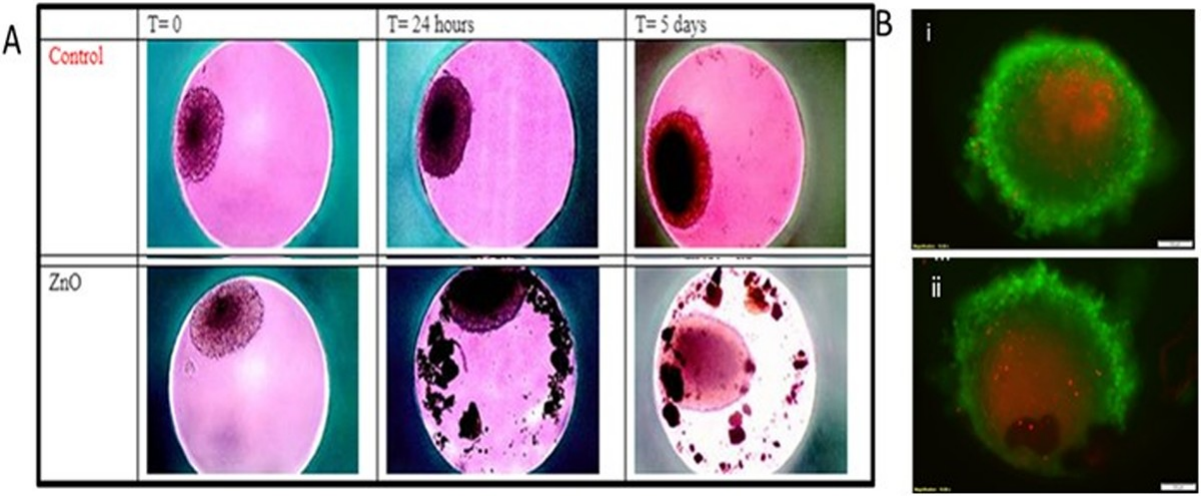
A. Anticancer activity of ZnO NP in HeLa cell spheroids at 0 h, 24 h and day. B. i. Untreated spheroids ii. Spheroids treated with ZnO NP.

3-D spheroids are important models for drug discovery and delivery to tumor [23]. HeLa cell spheroids were cultured and the spheroids allowed to form prior to nanoparticle treatment and were exposed to ZnO NP at 10 or 20 ug/ml concentration respectively in serum containing media and photographed at time 0, 24 and 5 days by light microscopy as shown in Fig 4A.

As seen in Fig 4A, overt growth inhibition was seen particularly after NP exposure for 5 days. In the case of ZnO NP, these structures appeared to break apart and the dense cell growth radiating out from the center of the spheroid was almost completely absent. The degree of disintegration was profound on day 5. The appearance of the treated spheroids was quite distinct in comparison to untreated controls.

The spheroids were also stained by intravital stain to assess the relative kill zone from treated versus untreated samples and representative images shown after visulaization by fluorescence microscopy seen in Fig 4B.

Clearly, the kill zone was significantly increased after exposure to ZnO NP (4.B.ii.) relative to untreated controls (4.B.i.). These data are consistent with our recent work demonstrating intratumoral injection of ZnO NP in experimental mouse melanoma model also has significant antitumor activity *in vivo* [24].

### Zinc oxide combined with RBD exhibited anticancer activity in mouse melanoma cells

**Fig 5:**
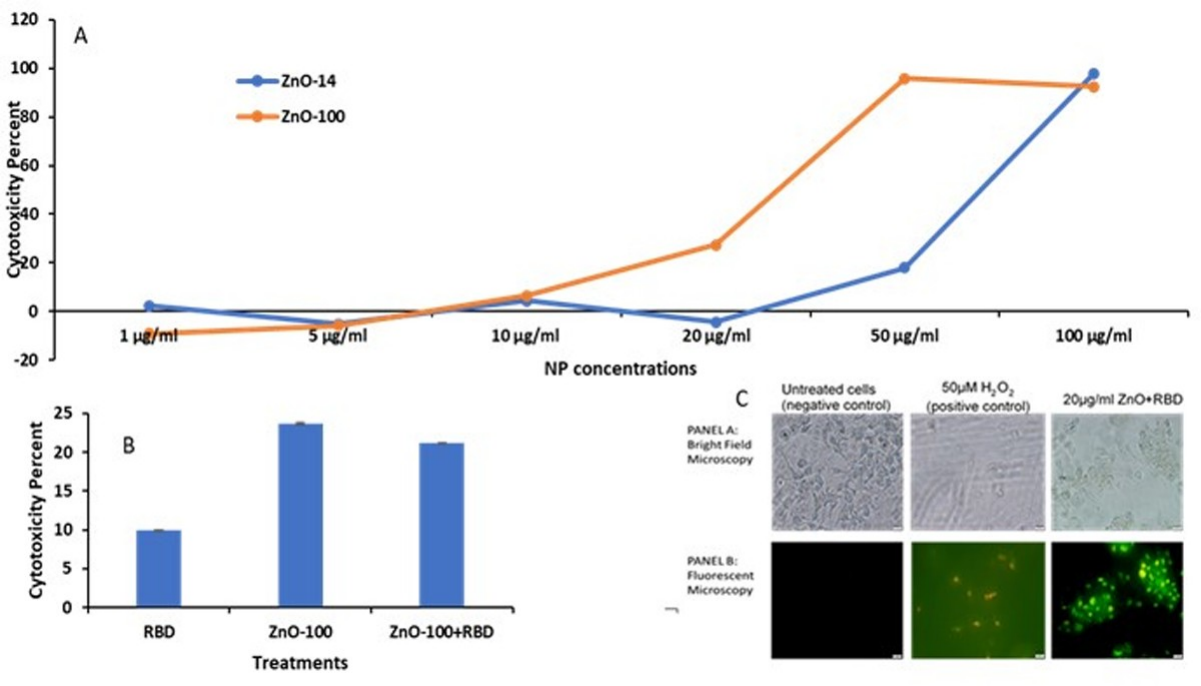
A. Comparative cytotoxicity in B16F10 cells in response to increasing doses of ZnO NP-100 nm and ZnO NP–14 nm B. Comparative cytotoxicity in B16F10 cells in response to RBD-bound ZnO NP 100 nm @ 20 μg/ml C. Bright field microscopic images (panel A) and fluorescence microscopic images (panel B) of B16F10 cells after 24 h exposure to ZnO + RBD.

Response of mouse melanoma cells to ZnO NPs, and RBD (both in the presence and absence of nanoparticle carriers) was measured in terms of cell viability using the colorimetric MTT assay. MTT assay measures the reduction of the water-soluble yellow tetrazolium salt 3-(4,5-Dimethylthiazol-2-yl)-2,5-diphenyltetrazoliumbromide into the insoluble purple formazan compounds by mitochondrial activity [25]. The total mitochondrial activity is directly related to the number of viable cells for most cell populations [26]. As evident from Fig 5A, the anticancer activity of ZnO NP–100nm was higher than ZnO NP–14 nm at 10, 20 and 50 μg/ml. Fig 5B shows that anticancer activity of RBD itself in the absence of NP was minimal. However, when the RBD was complexed to ZnO NP to form RBD-ZnO NP, it was quite potent with > 50% cytotoxic activity at 10 μg/mL dosage. In our previous work, we have shown that normal non-malignant cells treated with this concentration are normally 80–90% viable under these conditions. Cell viability was found to be reduced to 89% at 50 μg/mL RBD-ZnO.

### Zinc oxide-RBD complexes induce apoptosis morphology in mouse melanoma cells

Based on the cytotoxic activity, we were interested in visualizing the ZnO NP—induced apoptosis in mouse melanoma cells. B16F10 cells were treated with washed nanoparticles at a concentration of 0.05 mg/mL or 50μM H_2_O_2_ and imaged using an Olympus CKX41 Inverted Microscope after staining with Annexin-FITC / PI at 0 and 12 h [12, 13]. Fig 5C shows the cellular effects of ZnO-RBD treatment NPs with panel A showing bright field micrographs and panel B showing fluorescent micrographs. H_2_O_2_ served as the positive control causing apoptosis with many of the cells staining yellow/orange. Compared to the cells that didn’t receive RBD-ZnO, cell density was greatly reduced by H_2_O_2_ treatment. Some reduction of adherent cells was evident also after RBD-ZnO treatment indicative of cytotoxic activity, however these cells had a less spindle and more rounded morphology typical of apoptotic cells. These cells also stained yellow/orange consistent with their apoptosis.

In brief, the data demonstrate interaction of RBD to ZnO NP. In this study, zeta potential measurements and elution of the protein followed by gel electrophoresis analysis support formation of RBD-ZnO complexes. Fluorescence quenching by spectroscopy experiments shows the protein interaction to ZnO NP, which is important for ZnO NP mediated intracellular protein delivery. ZnO NP can inhibit 3-D tumor spheroid phenotype and growth demonstrating a loss of internal cell density and a greater zone of killing redtogreen ratio than the untreated spheroids. ZnO NP particle size is stable in cell culture media and no evidence of substantial ion generation was observed. These data suggest ZnO NP can serve as a protein carrier to deliver RBD into the cells. The fact that RBD itself is non-cytotoxic, but RBD-ZnO gave dose-dependent anticancer activity strongly supports this. Indeed, RBD-ZnO also triggered overt cancer cell apoptosis as evident by the treated cell morphology and also apoptosis specific staining and fluorescence microscopy analysis. The impact of this research is substantial given the importance of the RAS pathway to a variety of difficult to treat cancers, and the lack of a RAS-specific drug. Therefore, nanoparticle delivery of intracellular RBD may serve as *protein interference*–a novel strategy to inhibit the critical RAS pathway and may have potential as an experimental therapeutic approach against melanoma and potentially other RAS-dependent cancers.

## Supporting information

S1 Dataset (XLSX)

S1 Fig (TIF)

## Abbreviations

NP: Nanoparticle
Kd: Dissociation constant
MAPK: Mitogen activated protein kinases
ERK: Extracellular signal activated kinases
