# Supplementary figures and images for: Interaction of Ras Binding Domain (RBD) by chemotherapeutic zinc oxide nanoparticles: Progress towards RAS pathway protein interference
Source: PLoS One. 2020 Dec 16;15(12):e0243802. doi: 10.1371/journal.pone.0243802 (PMC7744048; doi:10.1371/journal.pone.0243802)

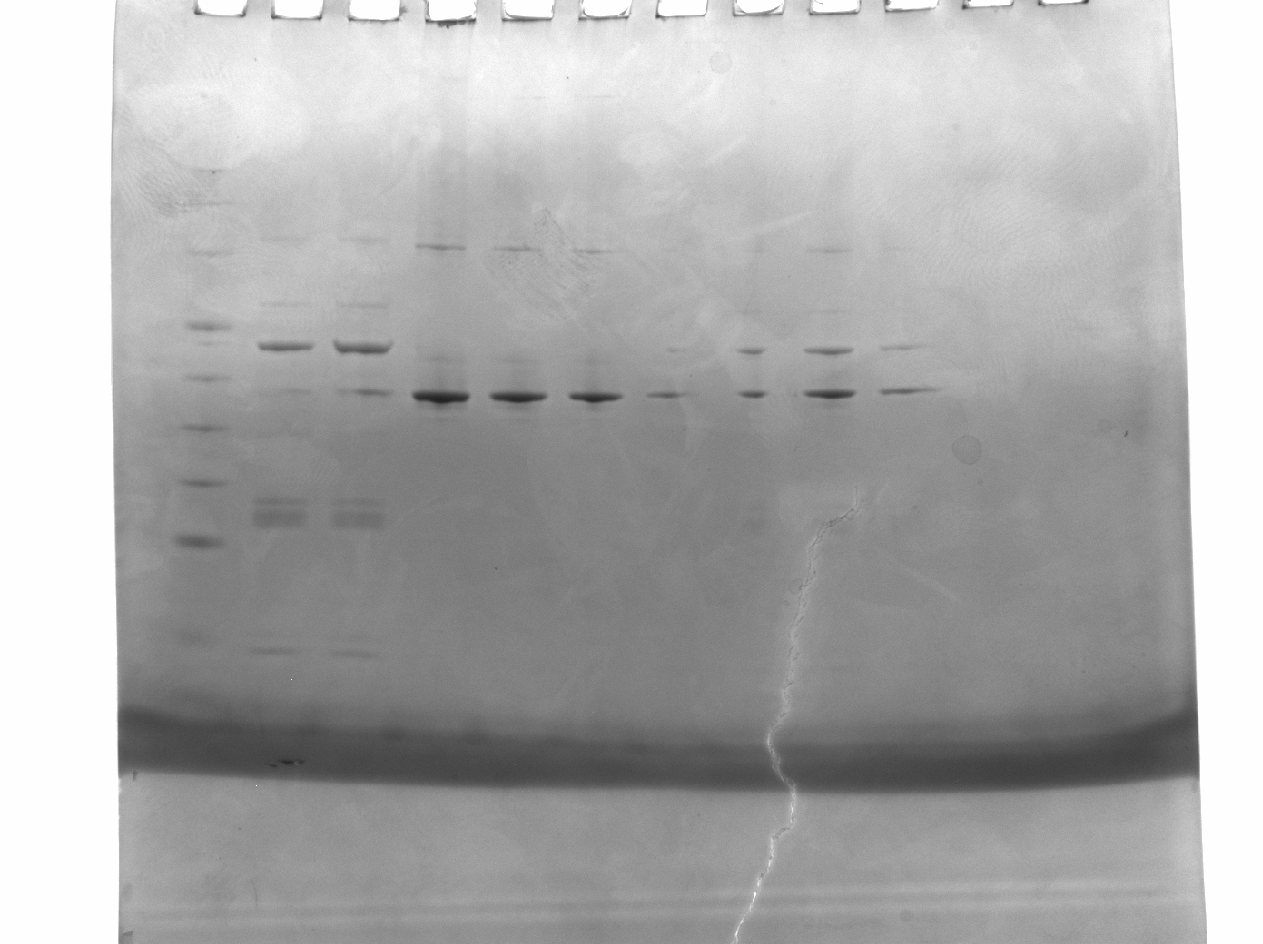

Supplement: S1 Fig — (TIF) [file pone.0243802.s002.tif]
